# T^2^W-CogLoadNet: a framework for cognitive load assessment of dance movements based on deep learning-powered human pose estimation

**DOI:** 10.3389/fpsyg.2025.1707539

**Published:** 2026-01-21

**Authors:** Fei Zhao

**Affiliations:** Shenyang Conservatory of Music, Dance Academy, Shenyang, Liaoning, China

**Keywords:** deep learning, dance, human pose estimation, Transformer, movement science, cognitive load assessment

## Abstract

Dance posture estimation and cognitive load assessment are crucial for optimizing dance training outcomes and promoting rehabilitation applications. Traditional methods often suffer from problems such as reliance on subjective judgment in cognitive load assessment and insufficient modeling of dance temporal features. This study proposes a T^2^W-CogLoadNet model, which integrates Temporal Convolutional Network (TCN)-Transformer temporal feature extraction with Whale Optimization (WOA) hyperparameter optimization to achieve 3D dance posture estimation and cognitive load modeling (indirect measurement). In this model, TCN captures local dynamic details of dance movements, Transformer handles long-range temporal dependencies, and WOA simultaneously optimizes feature subsets and model parameters to improve performance. Experimental validation on the AIST++ professional dance dataset and the Kinetics 400 generalized motion dataset demonstrates that the model significantly outperforms baseline models such as High Resolution Network (HRNet) and OpenPose estimation. On the AIST++ dataset, its mean absolute error (MAE) for cognitive load estimation reaches 0.23, root mean square error (RMSE) reaches 0.26, and mean mean joint error (MPJPE) for 3D joints reaches 0.45. On the Kinetics 400 dataset, MAE, RMSE, and MPJPE reach 0.25, 0.28, and 0.48, respectively. Even under interference scenarios such as noise injection and temporal scaling, the model maintains robust performance, with its MAE consistently lower than the aforementioned baseline models. Future research will focus on integrating multimodal inputs to improve assessment reliability, enhancing the model's adaptability to different dance styles, and developing lightweight real-time monitoring tools to promote the widespread application of this technology in dance education and rehabilitation.

## 1 Introduction

Dance, as a complex activity that integrates motor control, cognitive coordination, and emotional expression, has become a hot topic in the interdisciplinary fields of sports science, cognitive neuroscience, and psychology (Castro et al., 2024). Existing studies have confirmed that dance not only enhances cognitive abilities such as spatial memory and attention distribution through body coordination training, but also serves as a non-pharmacological intervention to improve emotional regulation in patients with depression and executive function in individuals with cognitive impairments (Rani and Devarakonda, 2022). However, these studies mainly focus on the “macro effects” of dance on cognition, while exploration of the “mechanism by which dance movement features correlate with cognitive load” remains insufficient (Hackney et al., 2024; Cheng et al., 2025). Cognitive load, as a core indicator reflecting the consumption of cognitive resources, is a key prerequisite for optimizing dance teaching (such as dynamically adjusting the difficulty of movements) and designing dance therapy (such as matching low-load movements to patients) through objective assessment (Liu and Ko, 2022; Sun and Wu, 2023) However, the current mainstream assessment methods still rely on subjective scoring tools such as the NASA TLX scale, which calculate cognitive load by weighting scores of dimensions such as psychological needs, physiological needs, and time needs (Aitsam et al., 2024). This not only has the limitations of large individual subjective bias and inability to conduct real-time dynamic assessment, but also lacks a clear correspondence between kinematic characteristics and cognitive load dimensions. There is an urgent need to establish a quantitative assessment system based on objective data.

With the development of computer vision and deep learning technologies, human pose estimation has provided a new pathway for objective analysis of dance movements (Matsuyama et al., 2021; Hu and Ahuja, 2021). In existing studies, HRNet with its high-resolution feature maps has the advantage of capturing subtle joint movements, such as finger coordination and ankle rotation, in dance actions (Li et al., 2024; Huang et al., 2025). Its 3D pose output ability has been used for difficulty classification of ballet, hip-hop, and other movements (Li Q. et al., 2022). While OpenPose is strong in generalization and real-time performance for 2D pose estimation, it lacks precision in the spatial and three-dimensional reconstruction of dance movements (e.g., the body extension angle in arabesque movements), making it insufficient for extracting key features related to cognitive load (Mroz et al., 2021; Jo and Kim, 2022). Building on this, temporal deep learning models have further advanced the study of the “movement feature-cognition correlation.” TCN (Temporal Convolutional Network), with its causal and dilated convolutions, efficiently captures short-term dynamic features of dance movements (e.g., sudden joint angle changes, body coordination speed) and has been used for real-time cognitive demand analysis in running and yoga (Cao, 2024; Qi and Sun, 2025). However, it is limited by its receptive field and cannot model the logical coherence of action segments in long sequences (e.g., the connected sequence of “tiptoe-spin-landing” in ballet). Transformer, with its self-attention mechanism, can capture long-range temporal dependencies of dance movements (e.g., cumulative effects of high-difficulty movements within a minute) and outperforms traditional RNNs in predicting cognitive load trends for gymnastic movements (Xu et al., 2022; Büyükgökoğlan and Uğuz, 2025). However, it is less sensitive to local movement suddenness (e.g., quick hand spins in street dance), and may overlook critical features of immediate cognitive load. LSTM and its variants (e.g., BiLSTM) can handle temporal continuity but suffer from gradient vanishing issues, causing significant performance degradation in analyzing long sequences of dance movements (e.g., modern dance over 5 min) (Mu, 2022). Furthermore, existing research has not clearly defined the correspondence between core motor attributes and cognitive load dimensions. For example, “coordination entropy” (which quantifies the complexity of multi-joint coordination by the information entropy of the motion trajectories of 25 key joints) can be used as a proxy indicator for “motor control requirements.” The higher the value, the worse the synchronicity of joint motion, and the more cognitive resources are required for coordination. The lack of such theoretical mapping leads to a lack of solid logical support for cognitive load assessment.

To address these deficiencies, this study proposes a cognitive load assessment model for dance movements, T^2^W-CogLoadNet, which integrates TCN, Transformer, and Whale Optimization Algorithm (WOA). It constructs an end-to-end framework with the process “3D pose estimation → temporal feature extraction → dual-dimension optimization → cognitive load quantification.” The innovative points of this research are as follows:

- Innovation in temporal feature fusion: A “TCN + Transformer” dual-module collaborative structure is designed. TCN captures local instantaneous features of dance movements (e.g., joint angle change rates), while Transformer models the cumulative correlations of long sequences (e.g., movement segment transitions). The dual modules complement each other, covering the full temporal dimension of dance movements and overcoming the limitations of single temporal models.
- Innovation in optimization mechanism: WOA is introduced to achieve “parameter-feature” dual optimization, simultaneously adjusting the hyperparameters (e.g., TCN dilation rate, Transformer attention head numbers) and global feature subsets of TCN and Transformer. This solves the problem of individual difference adaptation for different dancers/dance styles while eliminating redundant features to improve evaluation accuracy.
- Innovation in data and scene adaptation: The AIST++ professional dance dataset is used as the core, utilizing its 3D joint annotations and music synchronization information to match the scene-specific needs of cognitive load assessment. Meanwhile, a pre-trained model using the Kinetics 400 dataset is employed to balance generalization and scene-specific adaptation. This study establishes the first quantifiable mapping between “3D dance pose features” and “cognitive load.”

The subsequent sections are organized as follows: Section 2 “Related Work” systematically reviews the progress in dance cognitive load assessment, human pose estimation, and temporal deep learning models, further highlighting the research gap. Section 3 “Method” details the overall architecture, module design, experimental data preprocessing, and evaluation metrics of T^2^W-CogLoadNet. Section 4 “Experiments” validates the model's performance and the effectiveness of its innovations through loss analysis, ablation experiments, comparative experiments, and robustness tests. Section 5 “Conclusion and Future Directions” summarizes the research findings, analyzes theoretical and practical value, and points out the research limitations and future expansion directions.

## 2 Related work

### 2.1 Dance cognitive load assessment and human pose estimation

Dance cognitive load assessment serves as the key link connecting dance movement features with the consumption of cognitive resources in the brain. Existing assessment methods are mainly divided into subjective and objective approaches. Subjective assessments primarily rely on questionnaires and rating scales, among which the NASA-TLX scale is the most widely used. It calculates overall cognitive load by weighted scoring across six dimensions, including mental demand, physical demand, and temporal demand (Kirakosian et al., 2021). This method is simple to operate and can directly reflect the dancer's subjective experience, and has been applied to load assessment in scenarios such as basic ballet training and street dance choreography (Shynar et al., 2024). However, this approach depends on the dancer's subjective judgment. Different dancers have varying perception thresholds for movement difficulty and standards for describing “effort level,” and it cannot capture the dynamic changes of cognitive load during movement execution in real-time, making it insufficient for fine-grained evaluation (Qi et al., 2022; Xing et al., 2025). Objective assessments focus on physiological signals and movement data. EEG (electroencephalography) can reflect the brain's attention allocation state through changes in α-wave power, while heart rate variability (HRV) can indirectly indicate the relationship between the autonomic nervous system and cognitive load (Li et al., 2023). Both have been used to analyze load fluctuations in complex dance movements, such as improvisational modern dance. However, physiological signals are easily affected by environmental noise and the dancer's emotional state, and it is difficult to directly establish a mapping between specific physiological changes and particular dance movement features, resulting in a research gap where “assessment results are disconnected from movement context” (Kodipalli et al., 2023).

The development of human pose estimation technology provides essential support for the objective quantification of dance movements, with deep learning models becoming the mainstream tools in this field. HRNet (High-Resolution Network), by maintaining high-resolution feature maps throughout its layers, can accurately capture subtle joint movements, such as finger coordination and ankle rotation, in dance actions (Li et al., 2024). Its 3D pose output achieves millimeter-level precision and has been used for difficulty grading in ballet arabesque and Latin dance steps, constructing scoring systems based on joint angles and limb extension parameters (Wang et al., 2024). In comparison, OpenPose demonstrates strong generalization and real-time performance in 2D pose estimation, allowing rapid processing of multi-person dance scenes, but lacks precision in reconstructing the spatial and three-dimensional relationships of movements (e.g., the spatial positions in freeze-frame street dance poses) (Mroz et al., 2021). GCNs (Graph Convolutional Networks), by modeling the human skeleton as a graph, enhance the learning of spatial correlations among joints and achieve over 95% accuracy in sports dance classification, but their capability to model temporal dynamics is limited (Zhang, 2025; Chen et al., 2024).

In summary, existing pose estimation models have respective advantages in extracting either “static spatial features” or “single-dimension dynamic features,” but none have yet developed a solution that can simultaneously adapt to both “dance movement precision” and “temporal continuity.” This provides guidance for the selection and optimization of the pose estimation module in this study—priority should be given to models that combine spatial accuracy with temporal adaptability, laying a data foundation for subsequent cognitive load correlation analysis.

### 2.2 Temporal deep learning and heuristic optimization algorithms

Temporal deep learning models are essential tools for analyzing the “temporal dimension features” of dance movements. Different models exhibit varying advantages in temporal modeling, and a single model is insufficient to cover the full range of temporal features required for dance movements (Li et al., 2025). TCN (Temporal Convolutional Network), with causal convolutions and dilated convolutions, can flexibly adjust the receptive field and efficiently capture the local dynamic features of dance movements in short windows, such as the rate of joint angle change and the collaborative variation of body parts between adjacent frames (Cao, 2024; Hao et al., 2025). It has been applied to real-time cognitive load correlation studies of yoga flow and basic dance steps. However, due to the limitation of its receptive field, TCN struggles to effectively model the logical connections between movement segments in long sequences, such as the coherent dependencies in ballet with “tiptoe - spin - landing” (Bai et al., 2025). Transformer, leveraging self-attention mechanisms, can model global dependencies in long-term dance movements, such as a 5-min folk dance combination, and identify high-load movement segments, such as complex turns involving multi-joint coordination (Xu et al., 2022). However, it lacks sensitivity to local movement suddenness, such as the key cognitive load features in fast hand turns in street dance (Zhao et al., 2025; Miao et al., 2025). RNNs (Recurrent Neural Networks) and their variants LSTM (Long Short-Term Memory) and GRU (Gated Recurrent Unit) are capable of processing temporal continuity in dance movement sequences through the memory unit characteristics (Büyükgökoğlan and Uğuz, 2025). LSTM, with its input, forget, and output gates, controls the flow of information, effectively addressing the vanishing gradient problem encountered by RNNs in long sequences (Mu, 2022). It has been applied in dance movement prediction and classification tasks, but its computational efficiency is low when dealing with long and complex dance movements, making it difficult to meet real-time requirements. GRU, with a simpler structure, operates faster, but it is less effective in capturing complex long-term dependencies (Bharathi et al., 2024). WaveNet, a specialized convolutional neural network, can generate high-quality audio and time-series data through dilated convolutions and residual connections (Valle-Pérez et al., 2021). It can simulate the fluidity of dance movements in dance movement generation but has less research on semantic understanding of movements and their correlation with cognitive load. Neural network-based time series decomposition models, such as N-BEATS and N-HiTS, perform excellently in time series prediction by decomposing dance movement sequences into different frequency components for analysis (Huang et al., 2024). However, they struggle to adapt to the complex and dynamic nature of dance movements. This current situation clearly demonstrates the urgent need for “dual-module fusion,” as only by complementing the advantages of different temporal models can we achieve full-dimensional coverage of dance movements' “local instantaneous features + long-range cumulative features.”

Heuristic optimization algorithms play an important role in tuning deep learning models. Different algorithms show significant differences in parameter optimization and feature selection efficiency. WOA (Whale Optimization Algorithm), an intelligent optimization algorithm that simulates the hunting behavior of whales, has the characteristics of fast convergence and strong global search capabilities (Hussain et al., 2022). Its “encircling prey-bubble net attack” optimization mechanism effectively avoids local optima and has been used for hyperparameter tuning in CNN-LSTM time series prediction models, reducing prediction errors by over 20% (Shi et al., 2025). In feature selection tasks, WOA can synchronize the removal of redundant features and noise interference through fitness function design. For example, in human action recognition, the key joint feature subset selected by WOA can reduce the model's computational load while improving classification accuracy. In contrast, PSO (Particle Swarm Optimization) is widely applied in parameter optimization but often suffers from convergence stagnation in the later stages of iteration (Shi et al., 2025). GA (Genetic Algorithm) maintains population diversity through crossover and mutation operations but has low optimization efficiency and poor adaptability to high-dimensional parameter spaces. For the needs of the dance cognitive load assessment model—requiring simultaneous optimization of “TCN-Transformer dual-module hyperparameters” and “pose feature subsets”—WOA's “dual-dimensional optimization capability” and “strong robustness” are more adaptable and can effectively address model performance fluctuations caused by differences in dancer movements and the distribution of features across different dance styles.

## 3 Methods and materials

### 3.1 Datasets and pre-processing

To ensure the “scene specificity” and “model generalization” of dance cognitive load assessment, this study adopts a dual-data support strategy with “core dataset + auxiliary dataset.”

The core dataset chosen is the AIST++ dataset, which is currently the largest and most finely annotated professional dance motion dataset (Tsuchida et al., 2019; Li et al., 2021). Its advantages align well with the research needs: First, the dance scenes are highly targeted, covering 10 typical dance types, including ballet, jazz, hip-hop, Latin, and others, with 500 action sequences (total duration of about 12.5 h, individual sequence duration ranging from 7.4 to 48.0 seconds). All sequences are performed by 20 professional dancers (aged 20–30, with a male-to-female ratio of 1:1, each with over 3 years of dance training experience), allowing for a diverse range of motion complexities across different dance styles (e.g., the body extension movements in ballet and the explosive quick movements in hip-hop), providing varied samples for cognitive load assessment. Second, the dataset offers rich annotation information, including 9 synchronized video views, 3D coordinates for 25 key joints (with annotation accuracy ≤ 2mm), and a music synchronization track with a 44.1 kHz sampling rate. The 3D joint data quantifies the spatial complexity of the movements (e.g., body extension angles, joint coordination), while the music information helps relate “rhythm synchronization” to cognitive load (e.g., fast-paced movements requiring higher attention allocation), addressing the lack of dance scene annotations in traditional general motion datasets.

The auxiliary dataset chosen is the Kinetics 400 dataset (Kay et al., 2017), from which 100 classes of movements related to “bodily dynamic motion” (e.g., yoga, gymnastics, running, ball sports) are selected, comprising 100,000 videos (each video lasting 10 seconds). This dataset is used for pre-training the model: First, it enhances the generalization of the pose estimation module, as this dataset includes bodily movements from various ages (18–45 years), body types, and movement scenes, with a gender ratio of approximately 52:48. It helps the HRNet model learn the feature patterns of “non-dance bodily movements,” indirectly improving the model's accuracy in capturing special joint variations in dance movements (e.g., ballet toe points, hip-hop hand spins). Second, it optimizes the temporal feature extraction module by pre-training on generalized motion time-series data, reducing the overfitting risk of the TCN-Transformer module on the AIST++ dataset and ensuring the model's adaptability to “non-standard dance movements” (e.g., non-standard movements from amateur dancers). Table 1 provides a comparison of the core features of the two datasets.

**Table 1:** Core feature comparison of AIST++ core dance dataset and kinetics 400 auxiliary movement dataset.

**Dataset**	**Data type**	**Movement categories/number**	**Key annotation information**	**Data scale**	**Research focus**
AIST++	Professional dance movements	10 dance types/500 action sequences	3D joint coordinates (25 Joints), 9 viewpoint videos, music tracks	Total duration 12.5 h	Core experimental data, supporting cognitive load quantification
Kinetics 400	General body movements	100 movement types/100,000 videos	2D action labels, video frames	10 seconds per video	Auxiliary pre-training data, enhancing model generalization

**Figure 1:**
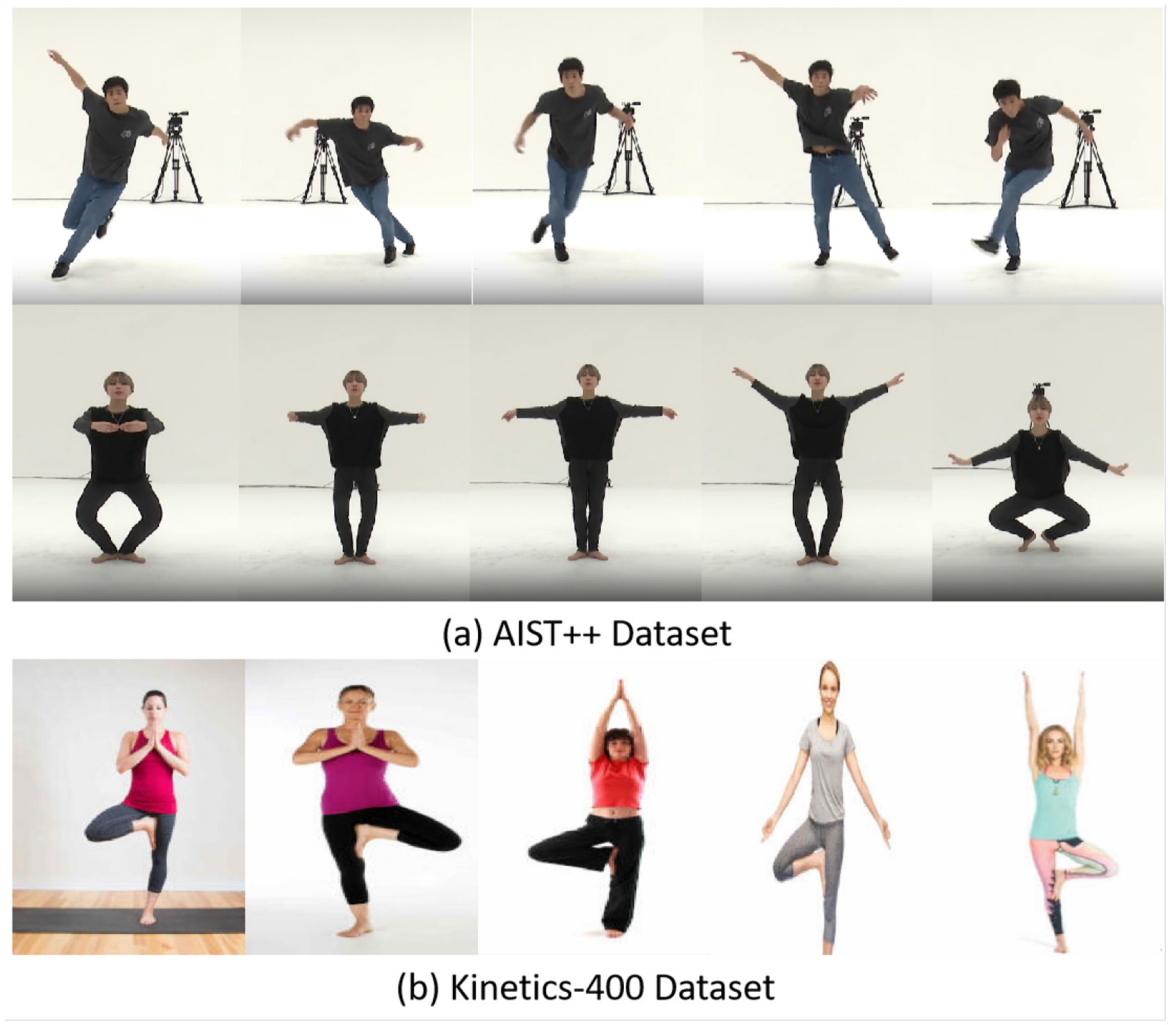
Typical sample images from the experimental datasets. **(a)** Dance movement samples from the AIST++ dataset; **(b)** “Tree Pose” movement sample from the Kinetics 400 dataset.

Figure 1 presents typical sample images from the AIST++ and Kinetics 400 datasets. The AIST++ dataset samples showcase diverse dance poses from different dancers, highlighting the richness and dynamics of dance movements. The “Tree Pose” sample from the Kinetics 400 dataset, representing generalized body movements, demonstrates commonality with dance movements in terms of limb coordination and can be used for pretraining the model to learn features of “static balance movements.”

The cognitive load true labels in this study were not constructed using subjective rating tools such as NASA-TLX, but were based on multi-source objective data to avoid the influence of subjective bias on the assessment results. We extracted professional dance annotation information from the AIST++ dataset, including the difficulty levels of 10 dance categories (levels 1–5, pre-annotated by 3 senior choreographers) and the collaborative complexity of 25 key joints (quantified by coordination entropy, calculated as $E=-\overset{25}{\underset{i=1}{\sum }}p_{i}\log_{2}p_{i}$, where *p*_*i*_ is the probability distribution of the motion trajectory of the *i*th joint). Next, combining synchronously acquired dancer physiological signals [the low-frequency/high-frequency ratio of heart rate variability (LF/HF) and the alpha wave power change rate of EEG], we determined the weights of each indicator using the analytic hierarchy process (AHP) (movement difficulty level 0.4, joint coordination entropy 0.3, LF/HF ratio 0.2, alpha wave power change rate 0.1), and constructed the cognitive load quantification formula: *y* = 0.4 × *D*+0.3 × *E*+0.2 × (*LF*/*HF*)+0.1 × Δα (where *D* is the difficulty level of the movement, and Δα is the rate of change of alpha wave power); finally, the quantification results are normalized to the [0,100] interval through min-max normalization as the true label of cognitive load. To verify the reliability of the label, three experts with more than 10 years of experience in dance teaching and movement analysis were invited to conduct a consistency evaluation of the label values of 100 randomly selected movement samples. The intragroup correlation coefficient (ICC) reached 0.92, indicating that the label has excellent reliability and rationality.

To eliminate data noise, standardize the input format, and enhance model generalization, a standardized preprocessing flow is designed for both the AIST++ and Kinetics 400 datasets. First, frames are extracted from the videos of both datasets at 25 fps. AIST++ uses the “gray variance threshold method” (frames with variance < 50 are considered blurry) to select clear frames (retaining 90% of the frames in 482 sequences). Kinetics 400 retains the core action frames from the middle 5 seconds to reduce redundancy. Then, the filtered frames from AIST++ are input into the pre-trained HRNet-W48 model to generate 3D coordinates for 25 key joints. The coordinates are mapped to the [0,1] range using min-max normalization (Equation 1), eliminating dancer height (1.55–1.85m) and body type differences, where *x* represents the original coordinate, and *x*_min_/*x*_max_ represent the minimum/maximum coordinate values for the corresponding joint in AIST++. Next, to meet the fixed sequence length requirement of Transformer (100 frames), short sequences (< 100 frames) from AIST++ are linearly interpolated, while long sequences (>100 frames) are segmented based on music beats. Kinetics 400 is directly segmented into 100-frame subsequences. Finally, a “5-frame sliding window Gaussian filter” (standard deviation = 0.8) is applied to suppress AIST++ coordinate noise, and three types of augmentation strategies are designed to simulate real dance variations: temporal scaling (±10% frame rate), spatial noise (±5% coordinate perturbation), and feature disturbance (±3°/frame joint angle deviation). After preprocessing, AIST++ sequences are split into training (337 sequences), validation (96 sequences), and test (49 sequences) sets, ensuring a consistent distribution of dance types across the datasets.


$$\begin{array}{l} x_{\text{norm}}=\frac{x-x_{\text{min}}}{x_{\text{max}}-x_{\text{min}}} \end{array}$$ (1)

### 3.2 Architecture of T^2^W-CogLoadNet model

**Figure 2:**
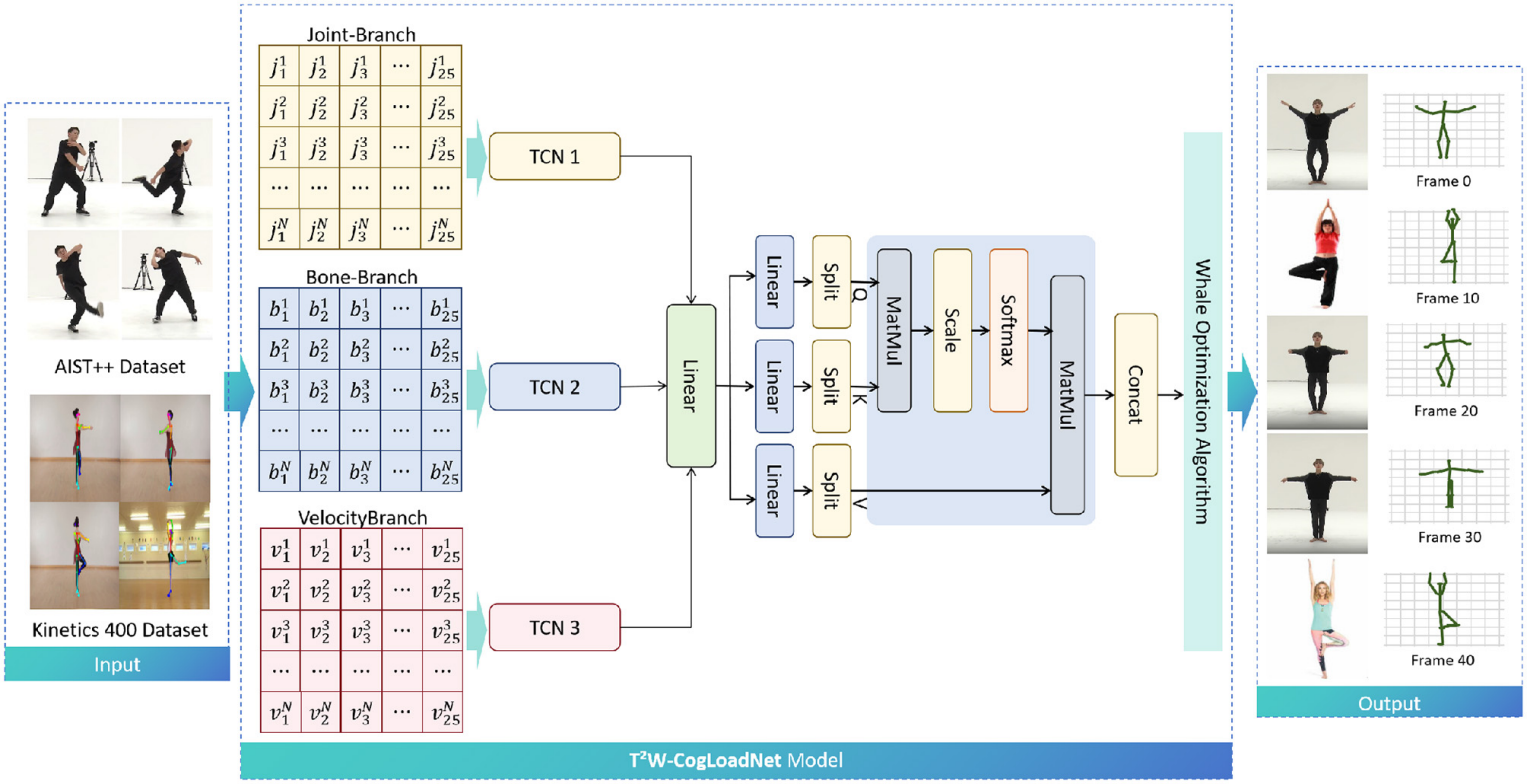
End-to-end architecture of T^2^W-CogLoadNet model from multi-source pose data to dance movement cognitive load.

The T^2^W-CogLoadNet model proposed in this study adopts a “four-module end-to-end” architecture. Through the full-process collaboration of “data input - feature extraction - optimization and control - load output,” it solves the problems of traditional models, such as “partial capture of dance features” and “insufficient robustness of cognitive load assessment.” The overall architecture and data flow of the model are shown in Figure 2.

From the input layer perspective, the core input to the model is a standardized 3D pose sequence obtained by pre-training HRNet-W48. The input data mainly comes from the AIST++ professional dance dataset (used for the entire model training and optimization process), while the Kinetics 400 dataset is only used for the pre-training of the HRNet-W48 backbone network (to improve the accuracy of general limb movement pose extraction). The 3D pose data generated by HRNet-W48 is further decomposed into Joint-Branch, Bone-Branch, and Velocity-Branch, forming an initial feature stream of “multi-branch parallel input,” which lays the foundation for subsequent temporal feature extraction.

In the core feature extraction layer, the features of the three branches are passed through Temporal Convolutional Networks (TCNs) for local temporal feature extraction: TCN 1 processes the 25 joint sequence data from the Joint Branch to capture “subtle changes in joint position over time” (e.g., the graceful wrist swinging trajectory in ballet); TCN 2 focuses on the skeletal topology sequence information from the Bone Branch to extract “spatial structural dynamic associations between body segments” (e.g., the linkage pattern between the torso and limbs in street dance); TCN 3 processes the joint velocity sequence from the Velocity Branch, focusing on “movement explosiveness and rhythm characteristics” (e.g., fast-slow transitions in Latin dance steps). The outputs of the three TCNs are then integrated through a linear layer, forming a “local temporal feature fusion vector,” which is further processed through multiple linear layers and Split operations, providing multi-dimensional feature inputs for the subsequent long-range association modeling.

In the long-range feature modeling and optimization layer, the fused features are input into an “improved Transformer structure” to achieve the mapping from “local features to long-range movement semantics”—using the attention mechanism to mine “cross-frame, cross-branch action logical associations” (e.g., the coherent sequential pattern of “raising hand - rotating - landing” in dance movements). Subsequently, the Whale Optimization Algorithm (WOA) is introduced to perform dual optimization on the feature weights output by Transformer. On one hand, it enhances the contribution of key action features, and on the other hand, it suppresses the interference of noisy features, improving the model's robustness to “individual differences among dancers and differences in dance styles.”

Finally, the features optimized by WOA are concatenated and linearly mapped to output the cognitive load assessment results of the dance movements, completing the end-to-end mapping from “multi-source pose data → temporal features → cognitive load.”

#### 3.2.1 Data input layer: dance movement 3D pose sequence construction

The data input layer is responsible for converting “multi-source video into standardized 3D pose sequences” through the process of “video frame processing - 3D coordinate generation - temporal and coordinate optimization,” providing high-quality input. First, video frames are extracted from the AIST++ and Kinetics 400 datasets at 25fps. A “grayscale variance threshold method” is used (frames with variance < 50 are considered blurry) to filter out clear frames, retaining sequences where the clear frame ratio is ≥ 90%. Then, the pre-trained HRNet-W48 model is used to generate the 3D coordinates for 25 key joints. For the Transformer's fixed sequence length requirement, sequences with fewer than 100 frames are completed using linear interpolation, while those with more than 100 frames are segmented according to musical beats. Finally, min-max normalization (mapping coordinates to the [0,1] range) and a 5-frame sliding window Gaussian filter (standard deviation = 0.8) are applied to suppress noise, outputting a uniformly long normalized 3D pose sequence as initial data for the model's multi-branch input.

#### 3.2.2 TCN module: local temporal feature extraction

**Figure 3:**
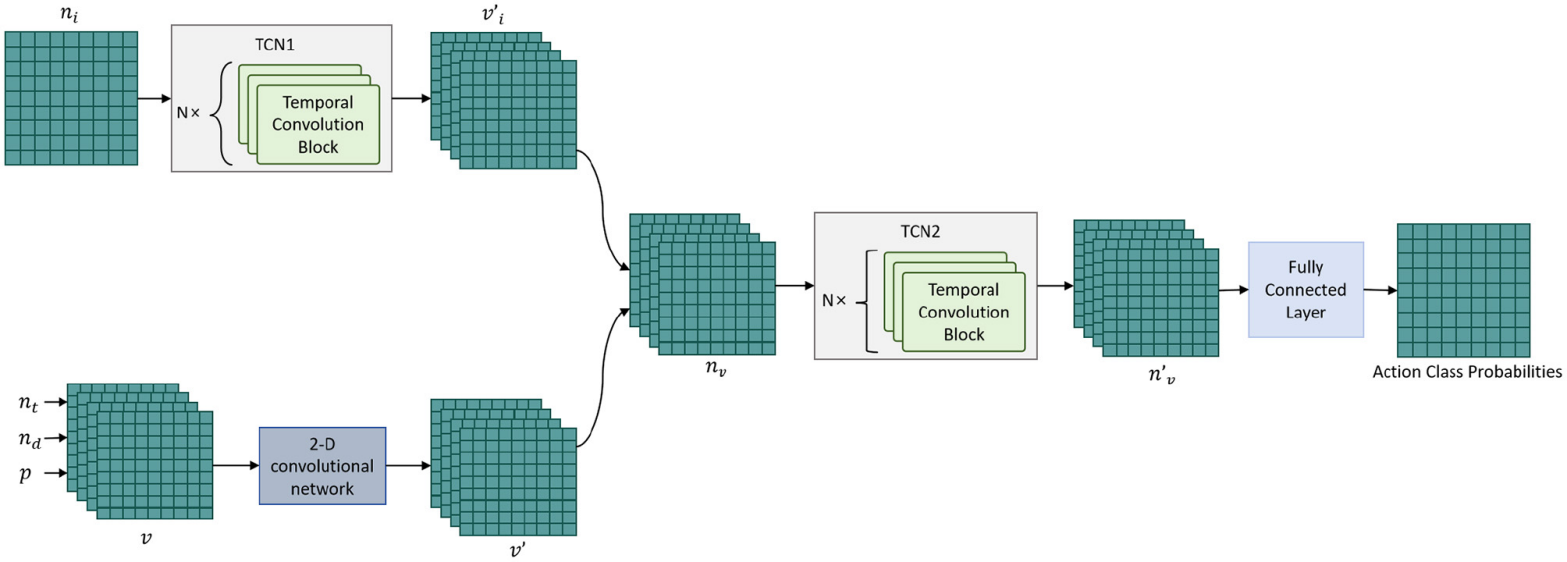
TCN module local temporal feature extraction process and structure.

The TCN module is the core component of the T^2^W-CogLoadNet model for capturing the “short-window dynamic details” of dance movements. It plays a key role in the conversion from “raw 3D pose sequences” to “interpretable cognitive load features.” It provides support for subsequent long-range temporal association modeling and cognitive load quantification, offering instantaneous dynamic features such as “joint angle change rates” and “limb coordination speed,” and serves as the core bridge linking “movement immediacy” with “instantaneous cognitive load.” The structure and data flow logic of the module are shown in Figure 3.

The TCN module aims to “capture the dynamic details within short windows (a continuous segment of movement frames)” of dance movements, focusing on local temporal features such as “joint angle change rates” (e.g., the change in elbow angle per second during ballet arm lifts) and “limb coordination speed” (e.g., the synchronized movement rate of limbs and torso in street dance). These features are directly related to the “instantaneous cognitive load” when the dancer performs the movement (e.g., fast, highly coordinated movements require higher attention allocation, corresponding to higher instantaneous load).

In terms of structure and parameter configuration, the TCN module implements local temporal feature extraction based on causal convolution principles. The computation for the *l*-th layer of causal convolution is given by:


$$\begin{array}{l} y_{t}^{l}=\overset{K-1}{\underset{k=0}{\sum }}w_{k}^{l}\cdot x_{t-d^{l}\cdot k}^{l-1} \end{array}$$ (2)

where $y_{t}^{l}$ is the output feature at time *t* for the *l*-th layer, *K* is the kernel size (set to *K* = 3 here), $w_{k}^{l}$ is the *k*-th weight of the convolution kernel for the *l*-th layer, and $x_{t-d^{l}\cdot k}^{l-1}$ is the input feature at time *t*−*d*^*l*^·*k* for the *l*−1-th layer. The *d*^*l*^ is the dilation rate for the *l*-th layer (taking values from the increasing sequence *d*^*l*^∈{1, 2, 4, 8, 16}), which controls the receptive field and adapts to different local temporal spans of dance movements.

The activation function used is LeakyReLU (Qi and Sun, 2025), defined as:


$$\text{LeakyReLU}(x)=\{\begin{array}{ll} x, & x\geq 0\\ \alpha x, & x<0 \end{array}$$ (3)

where α = 0.1 addresses the “neuron deactivation” issue in the negative gradient region of traditional ReLU, allowing for more accurate gradient flow for the subtle dynamic features of dance movements (e.g., the gradient of small joint angle changes).

The normalization operation uses LayerNorm, computed as:


$$\begin{array}{l} \text{LayerNorm}\left(x\right)=\gamma \cdot \frac{x-\mu }{\sqrt{\sigma^{2}+\epsilon }}+\beta \end{array}$$ (4)

where μ is the mean of the input feature *x*, σ^2^ is the variance, ϵ = 1*e*−5 is a small value to prevent division by zero, and γ and β are learnable scaling and offset parameters, which adapt to pose feature distribution differences across different dancers and dance styles, ensuring the comparability of features across different samples.

The TCN module adopts a 5-layer causal convolution structure. The receptive field gradually expands through the “increasing dilation rates (1 → 2 → 4 → 8 → 16)” design, allowing it to capture subtle joint changes between adjacent frames (with dilation rate 1, the receptive field covers 2 frames) and long continuous movement segments of up to 32 frames (with dilation rate 16, the receptive field covers 32 frames), adapting to the different local movement durations of various dance types (e.g., jazz dance's rapid tap movements are usually short frame windows, while modern dance's expansive movements often have long frame windows). Finally, after extracting local temporal features from the input standardized 3D pose sequence, the TCN module outputs a 256-dimensional local feature vector, providing the foundational input for subsequent long-range feature fusion and cognitive load assessment.

#### 3.2.3 Transformer module: long-range temporal association modeling

**Figure 4:**
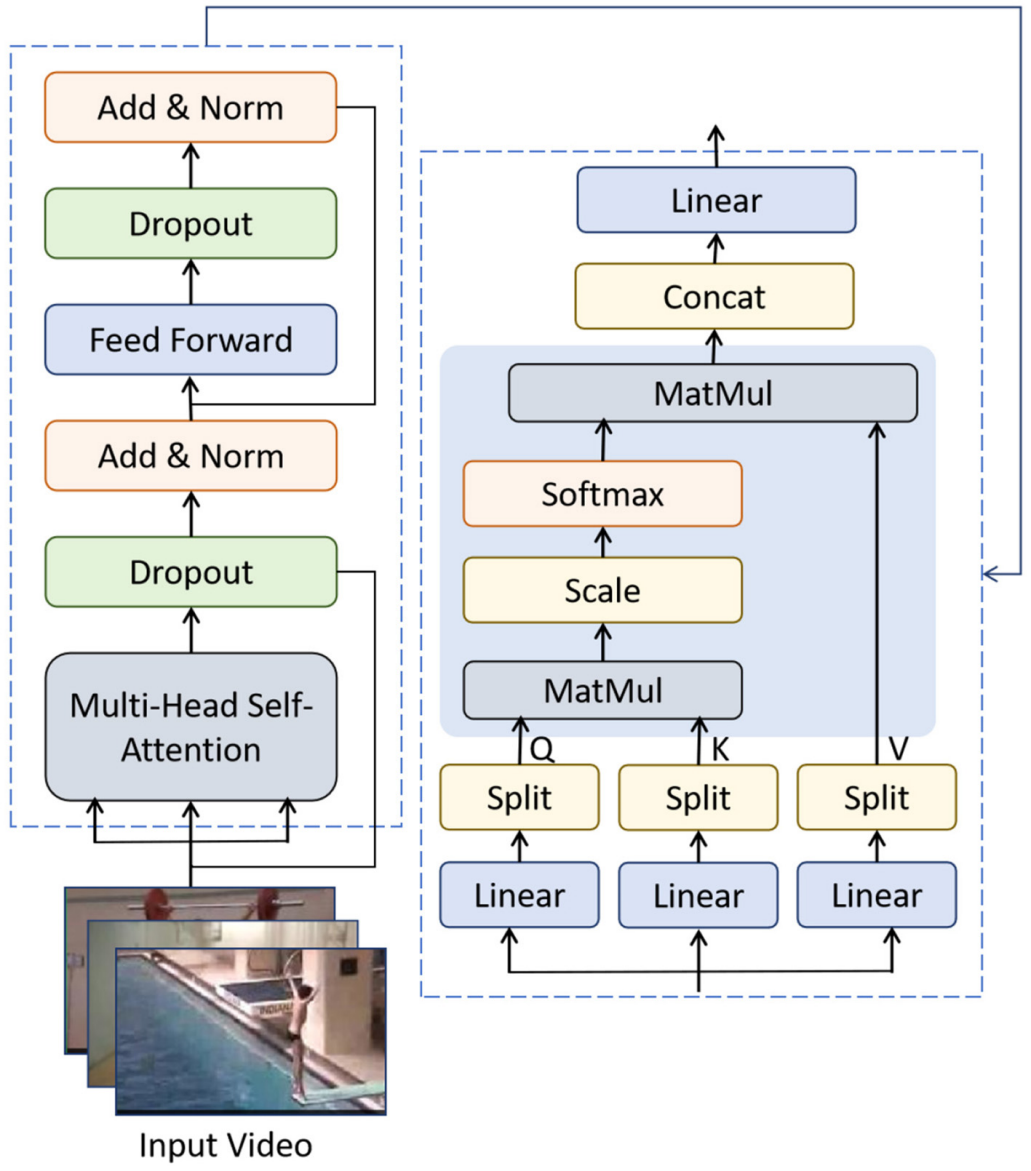
Transformer module long-range temporal association modeling structure and data flow.

The Transformer module is the core component of the T^2^W-CogLoadNet model for capturing “long-sequence logical associations” in dance movements. It plays a key role in upgrading from “local temporal features” to “global cognitive load patterns,” serving as the critical bridge between them. It receives the local features from the TCN module and extracts long-range associations such as “action segment transitions” and “rhythmic synchronization.” These associations directly correlate with the dancer's “cumulative cognitive load” during the execution of the entire movement.The structure and data flow of the module are shown in Figure 4.

The Transformer module aims to “capture the logical associations within long sequences (complete sequences made up of multiple continuous movement segments)” in dance movements. It focuses on long-range temporal features such as “action segment transitions” (e.g., the smooth transition from “arabesque” to “pirouette” in ballet) and “rhythmic synchronization” (e.g., the alignment between dance movements and music beats). These features are directly related to the dancer's “cumulative cognitive load” when performing a full sequence. More complex action segments and higher synchronization requirements typically result in higher cumulative cognitive load.

In terms of structure and parameter configuration, the core of the Transformer module is the multi-head self-attention mechanism (Li W. et al., 2022). The calculation for multi-head self-attention is given by:


$$\begin{array}{l} \text{MultiHead}\left(Q,K,V\right)=\text{Concat}\left({\text{head}}_{1},{\text{head}}_{2},\ldots ,{\text{head}}_{h}\right)W^{O} \end{array}$$ (5)

where *h* = 8 is the number of attention heads; ${\text{head}}_{i}=\text{Attention}\left(QW_{i}^{Q},KW_{i}^{K},VW_{i}^{V}\right)$, with $W_{i}^{Q},W_{i}^{K},W_{i}^{V}$ being the learnable weight matrices for the *i*-th head; *W*^*O*^ is the integration weight matrix for the multi-head output; and *Q, K, V* are the query, key, and value matrices, which are obtained by applying linear transformations to the local feature vectors output by TCN. These matrices capture the long-range associations between different frames and local features.

The attention score calculation (scaled dot-product mechanism) in self-attention is (Zhao et al., 2025):


$$\begin{array}{l} \text{Attention}\left(Q,K,V\right)=\text{Softmax}\left(\frac{QK^{T}}{\sqrt{d_{k}}}\right)V \end{array}$$ (6)

where *d*_*k*_ is the dimension of *K* (here *d*_*k*_ = 64, since the total dimension of multi-head self-attention is 512, and 512÷8 = 64), and $\sqrt{d_{k}}$ is used for scaling to prevent excessively large dot products that could lead to gradient vanishing in Softmax. This ensures more accurate calculation of the association weights between features at different temporal positions in the dance movements.

The feed-forward network uses the GELU activation function, defined as:


$$\begin{array}{l} \text{GELU}\left(x\right)=x\cdot \Phi \left(x\right) \end{array}$$ (7)

where Φ(*x*) is the cumulative distribution function of the standard normal distribution. Compared to ReLU, GELU provides smoother weighting of the input and is better suited to modeling nonlinear patterns such as “gradual intensity changes” and “rhythm transitions” in long dance sequences.

The Transformer module uses a 3-layer encoder structure, with each encoder containing the “multi-head self-attention + feed-forward network” submodules. It also incorporates “Add & Norm” (residual connections + layer normalization) and Dropout (to prevent overfitting) mechanisms. Through this structure, the Transformer module maps the 256-dimensional local feature vectors output by TCN to 512-dimensional global feature vectors, integrating long-range temporal association information from dance movements comprehensively, providing the global feature support for subsequent cognitive load quantification.

#### 3.2.4 WOA-driven dual optimization

**Figure 5:**
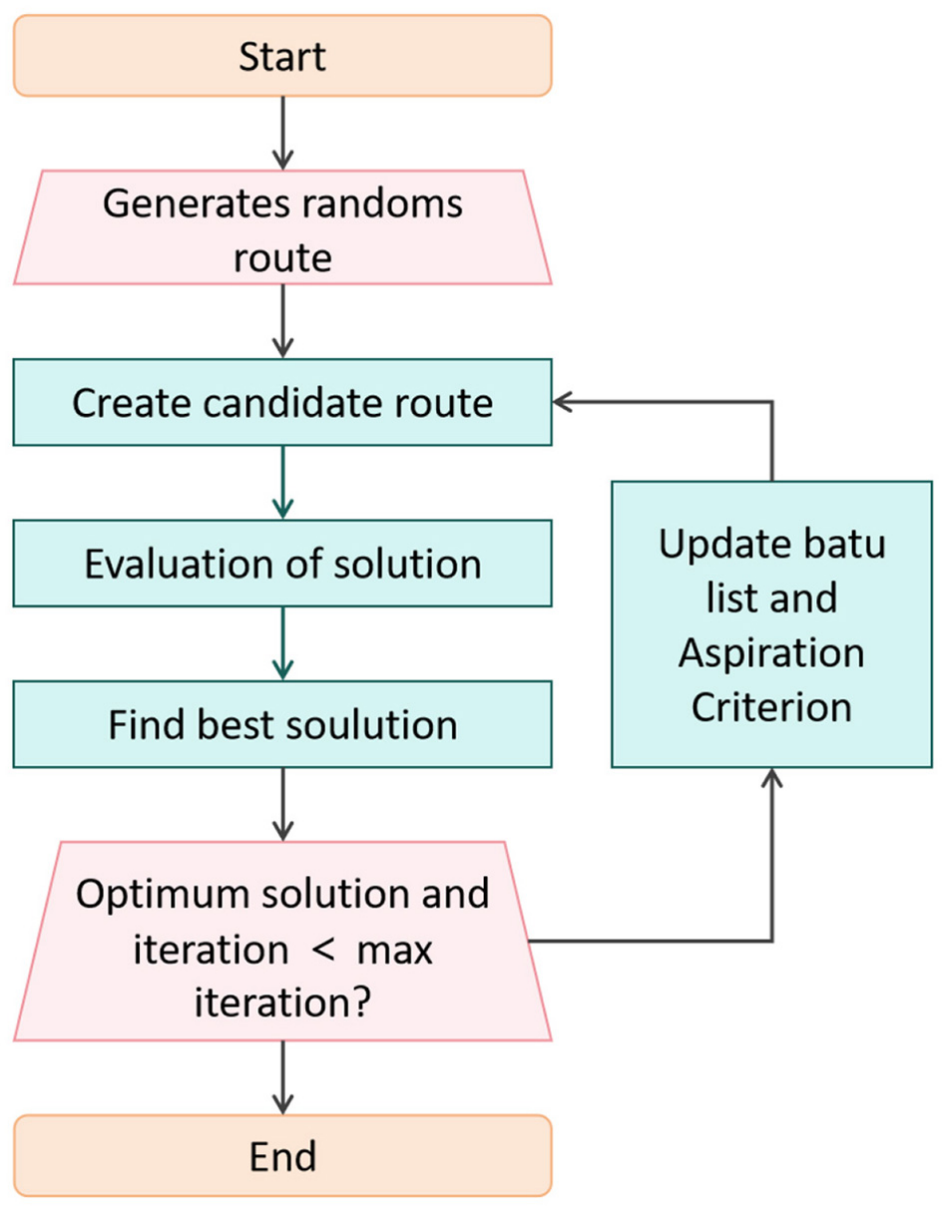
WOA-driven dual optimization process.

The optimization layer is the core guarantee for achieving “dual improvement in accuracy and efficiency” in the T^2^W-CogLoadNet model. It plays a decisive regulatory role in the iteration from “raw features and parameter combinations” to “optimal cognitive load assessment configuration.” Using the Whale Optimization Algorithm (WOA) as the core, it simultaneously optimizes the hyperparameters of TCN and Transformer, as well as the global feature subsets. It is the key to balancing “model complexity” and “cognitive load assessment accuracy.” The optimization process and logic are shown in Figure 5.

The optimization layer constructs a fitness function based on “Mean Absolute Error (MAE) + Feature Redundancy Coefficient (FRC)” to simultaneously pursue “high cognitive load assessment accuracy” and “low feature redundancy:”


$$\begin{array}{l} \text{Fitness}=\alpha \cdot \text{MAE}+\left(1-\alpha \right)\cdot \text{FRC} \end{array}$$ (8)

where α = 0.7 is the weight of MAE (emphasizing the core role of assessment accuracy); MAE is the mean absolute error between the model's predicted cognitive load values and the true values, computed as $\text{MAE}=\frac{1}{N}\overset{N}{\underset{i=1}{\sum }}|{ŷ}_{i}-y_{i}|$ (where *N* is the number of samples, ŷ_*i*_ is the predicted cognitive load, and *y*_*i*_ is the true cognitive load); FRC is the redundancy of the feature subset, computed as $\text{FRC}=\frac{1}{M^{2}}\overset{M}{\underset{j=1}{\sum }}\overset{M}{\underset{k=j+1}{\sum }}\text{Corr}\left(f_{j},f_{k}\right)$ [where *M* is the feature dimension, and Corr(*f*_*j*_, *f*_*k*_) is the correlation coefficient between the *j*-th and *k*-th features]. By reducing FRC, redundant features are eliminated to reduce interference with the model, improving the efficiency of the assessment.

WOA simulates three behaviors of whales (encircling prey, bubble-net attack, random search) to perform global and local optimization (Rajchowski et al., 2023). The position update of a whale is simulated to encircle the optimal solution, and the position update formula is:


$$\begin{array}{l} \vec{X}\left(t+1\right)={\vec{X}}^{*}\left(t\right)-\vec{A}\cdot |\vec{C}\cdot {\vec{X}}^{*}\left(t\right)-\vec{X}\left(t\right)| \end{array}$$ (9)

where $\vec{X}\left(t\right)$ is the position vector of the whale individual at the *t*-th iteration (corresponding to a set of TCN-Transformer hyperparameters and feature subsets); ${\vec{X}}^{*}\left(t\right)$ is the optimal position vector at the *t*-th iteration; $\vec{A}=2\vec{a}\cdot \text{rand}()-\vec{a}$, $\vec{C}=2\cdot \text{rand}()$ (where $\vec{a}$ linearly decreases from 2 to 0 across iterations, and rand() is a random number between [0,1]). The parameters $\vec{A}$ and $\vec{C}$ control the encircling range and direction, adapting to the optimization of hyperparameters such as TCN convolution layer number and Transformer attention head count, as well as feature subset encircling.

The bubble-net attack simulates the spiral approach of whales to prey. The position update formula is:


$$\begin{array}{l} \vec{X}\left(t+1\right)=|{\vec{X}}^{*}\left(t\right)-\vec{X}\left(t\right)|\cdot e^{bl}\cdot \cos\left(2\pi l\right)+{\vec{X}}^{*}\left(t\right) \end{array}$$ (10)

where *b* = 1 is a constant controlling the spiral shape, and *l* is a random number between [–1,1]. The spiral update enables fine search of the optimal hyperparameter combination and strongly correlated feature subsets, improving optimization accuracy.

When $|\vec{A}|\geq 1$, the whale performs a random search, with the position update formula given by:


$$\begin{array}{l} \vec{X}\left(t+1\right)={\vec{X}}_{\text{rand}}\left(t\right)-\vec{A}\cdot |\vec{C}\cdot {\vec{X}}_{\text{rand}}\left(t\right)-\vec{X}\left(t\right)| \end{array}$$ (11)

where ${\vec{X}}_{\text{rand}}\left(t\right)$ is the position vector of a randomly selected whale individual at the *t*-th iteration. The random search enlarges the exploration range of hyperparameter space (e.g., TCN dilation rate sequence, Transformer feed-forward network dimension) and feature space, avoiding convergence to local optima.

The optimization layer process is as follows: First, population initialization is performed, generating multiple combinations of TCN-Transformer hyperparameters and feature subsets. Then, “encircling prey/bubble-net attack/random search” position updates are sequentially executed to create candidate solutions. The candidate solutions are then evaluated for fitness (calculating MAE and FRC). The optimal solution is selected, and it is checked whether the “optimal solution is stable and the iteration count is less than the maximum iteration count (set to 100).” If not, the taboo list is updated (to avoid redundant searches for invalid solutions) and the desirability level (allowing the re-selection of high-quality solutions that were previously forbidden) is adjusted, continuing the iteration until convergence. The final output is the optimal TCN-Transformer hyperparameters and 300-dimensional strongly correlated features, providing accurate and efficient input for the cognitive load output layer.

#### 3.2.5 Cognitive load output layer: quantitative mapping

The cognitive load output layer is the “result terminal” of the T^2^W-CogLoadNet model, responsible for accurately mapping the 300-dimensional strongly correlated features output from the optimization layer to the dance movement cognitive load score on a scale of [0, 100]. This layer adopts a 3-layer fully connected network architecture, sequentially performing linear transformations from dimensions 300 → 128, 128 → 64, and 64 → 1, gradually compressing the feature dimensions and focusing on the core cognitive load information. Finally, the output is mapped to the target range through a Sigmoid activation function. The computation is given by:


$$\begin{array}{l} \text{Score}=100\cdot \text{Sigmoid}\left(W_{3}\cdot \text{ReLU}\left(W_{2}\cdot \text{ReLU}\left(W_{1}\cdot x+b_{1}\right)+b_{2}\right)+b_{3}\right) \end{array}$$ (12)

where *x* is the 300-dimensional feature vector output by the optimization layer; *W*_1_, *W*_2_, *W*_3_ are the weight matrices of the fully connected layers; *b*_1_, *b*_2_, *b*_3_ are the bias terms; ReLU is the rectified linear unit function (ReLU(*z*) = max(0, *z*)), introducing non-linearity to enhance feature expression capability; the Sigmoid function compresses the intermediate output to the range [0,1], and then it is multiplied by 100 to obtain the cognitive load score in the range of [0, 100].

To ensure the accuracy of high-load dance movements (e.g., complex ballet variations, rapid street dance combinations, which consume more cognitive resources and are prone to training bias due to evaluation errors), the loss function uses a weighted mean squared error (MSE), calculated as:


$$\begin{array}{l} \text{Loss}=\overset{N}{\underset{i=1}{\sum }}w_{i}\cdot {\left({ŷ}_{i}-y_{i}\right)}^{2} \end{array}$$ (13)

where *N* is the number of samples; ŷ_*i*_ is the predicted cognitive load score; *y*_*i*_ is the true score; and *w*_*i*_ is the weight for the *i*-th sample. When *y*_*i*_≥70 (high-load threshold), *w*_*i*_ = 1.5; otherwise, *w*_*i*_ = 1.0. By increasing the error weight for high-load movements, the model focuses more on fitting the accuracy of these movements during training, ensuring that the final evaluation results are applicable to high-load dance scenarios.

### 3.3 Experimental setup and parameter

In this study, an experimental environment suitable for complex temporal modeling and 3D pose feature processing is built, and key parameters for each module are determined through a “validation set grid search” with the goal of minimizing the mean absolute error (MAE) of cognitive load assessment on the validation set. The parameter ranges (e.g., TCN convolution kernel size, WOA population size) are tested in over 5 rounds, and the optimal parameter combination, balancing both “evaluation accuracy” and “computational efficiency,” is selected to provide the foundational support for model performance.

The experimental hardware is a high-performance server supporting multi-GPU parallel computing to meet the memory requirements for large-batch data training (e.g., 32 batch size 3D pose sequences) and complex modules (e.g., Transformer encoder). The specific configuration is as follows: CPU: Intel Xeon Gold 6,248 (20 cores, 40 threads, 2.5 GHz), GPU: 2 × NVIDIA Tesla V100 (32 GB HBM2 memory per card, supporting CUDA 11.8 parallel computing), Memory: 128 GB DDR4 (2,933 MHz), Storage: 2 TB SSD (550 MB/s read speed, used for dataset and model weight storage). The software environment is based on a Linux system, ensuring compatibility with all dependencies and efficiency in temporal data processing. Specifically, the operating system is Ubuntu 20.04 LTS, the deep learning framework is PyTorch 2.0.1 (with CuDNN 8.6 to accelerate convolution operations), the pose estimation libraries are OpenCV 4.8.0 (for video frame processing) and the official HRNet open-source library (for 3D joint coordinate generation), the optimization algorithm library is a custom WOA implementation based on NumPy 1.24.3, and the visualization tools are Matplotlib 3.7.1 (for result plotting) and TensorBoard 2.13.0 (for training monitoring).

Model parameters are categorized into “core feature extraction module (TCN/Transformer) - optimization module (WOA) - overall training,” with the parameter values and tuning rationale presented in Table 2.

**Table 2:** Key parameter settings for T^2^W-CogLoadNet model training for TCN, Transformer, WOA modules, and overall training (based on validation set tuning).

**Module type**	**Parameter name**	**Parameter value**
TCN module	Number of convolution layers	5 layers
	Convolution kernel size	3 × 1 (temporal convolution)
	Dilation rate sequence	[1,2,4,8,16]
	Activation function/normalization	LeakyReLU/ LayerNorm
Transformer module	Number of encoder layers	3 Layers
	Attention heads/hidden layer dimension	8 heads/512 dimensions
	Feed-forward network dimension	512 → 2048 → 512
	Activation function	GELU
WOA module	Population size/iterations	30/50
	Random search probability	10%
Training parameters	Initial learning rate	1e-4
	Batch size	32
	Training epochs/early stopping epochs	100 epochs/5 epochs
	Optimizer/weight decay	AdamW/1e-4

### 3.4 Metrics

This study selects five evaluation metrics: MAE, MSE, MPJPE, PCK, and AUC. Among them, MPJPE and PCK focus on the accuracy of joint positions in pose estimation, MAE and MSE address the overall bias and sensitivity to extreme errors in cognitive load prediction, and AUC measures the model's ability to distinguish between “high/low cognitive load movements,” forming a multi-dimensional and complementary evaluation system.

MAE is used to quantify the average deviation between predicted cognitive load values and true values, and is robust to outliers. The formula is:


$$\begin{array}{l} \text{MAE}=\frac{1}{N}\overset{N}{\underset{i=1}{\sum }}|{ŷ}_{i}-y_{i}| \end{array}$$ (14)

where *N* is the total number of dance movement samples; ŷ_*i*_ is the predicted cognitive load value for the *i*-th movement (ranging from [0,100]); and *y*_*i*_ is the true cognitive load value for the *i*-th movement. Based on the music beat annotations (44.1kHz sampling rate) and motion segmentation information of the AIST++ dataset, the preprocessed standardized 3D pose sequence of 100 frames (corresponding to 4 seconds, 25fps) is matched with the objective quantization load value within the same time period to ensure that each dance motion segment corresponds to a unique cognitive load true label, thus solving the problem of ambiguous temporal correspondence.

RMSE amplifies the influence of high-bias samples, highlighting extreme errors in cognitive load prediction. The formula is:


$$\begin{array}{l} \text{RMSE}=\sqrt{\frac{1}{N}\overset{N}{\underset{i=1}{\sum }}{\left({ŷ}_{i}-y_{i}\right)}^{2}} \end{array}$$ (15)

The variable definitions are the same as in MAE. A smaller RMSE value indicates higher prediction accuracy for high-load movements (e.g., complex ballet spins).

MPJPE evaluates the 3D joint position accuracy of the pose estimation module, directly reflecting the accuracy of skeletal modeling in dance movements. The formula is:


$$\begin{array}{l} \begin{array}{c} \text{MPJPE}=\frac{1}{N\cdot K}\overset{N}{\underset{i=1}{\sum }}\overset{K}{\underset{k=1}{\sum }}\\ \sqrt{{\left(x_{i,k}^{\text{pred}}-x_{i,k}^{\text{gt}}\right)}^{2}+{\left(y_{i,k}^{\text{pred}}-y_{i,k}^{\text{gt}}\right)}^{2}+{\left(z_{i,k}^{\text{pred}}-z_{i,k}^{\text{gt}}\right)}^{2}} \end{array} \end{array}$$ (16)

where *K* = 25 is the number of key joints annotated in the AIST++ dataset; $\left(x_{i,k}^{\text{pred}},y_{i,k}^{\text{pred}},z_{i,k}^{\text{pred}}\right)$ are the predicted 3D coordinates for the *i*-th movement and *k*-th joint; and $\left(x_{i,k}^{\text{gt}},y_{i,k}^{\text{gt}},z_{i,k}^{\text{gt}}\right)$ are the true 3D coordinates for the corresponding joint. All units are in meters (m).

PCK measures the “acceptable deviation rate” for pose estimation joint positions, adapting to the actual precision requirements for dance movements. The formula is:


$$\begin{array}{l} \text{PCK}\left(\tau \right)=\frac{1}{N\cdot K}\overset{N}{\underset{i=1}{\sum }}\overset{K}{\underset{k=1}{\sum }}I\left(\frac{d_{i,k}}{L_{i}}\leq \tau \right)\times 100\% \end{array}$$ (17)

where *d*_*i, k*_ is the Euclidean distance between the predicted and true positions of the *k*-th joint in the *i*-th movement; *L*_*i*_ is the diagonal length of the torso in the *i*-th movement (to eliminate height differences); τ = 0.1 is the deviation threshold (commonly used in dance movement evaluations); and *I*(·) is the indicator function, taking a value of 1 when the condition is met and 0 otherwise.

The Area Under the Curve (AUC) of the Receiver Operating Characteristic (ROC) evaluates the model's ability to classify “high/low cognitive load movements.” The formula is:


$$\begin{array}{l} \text{AUC}=\frac{1}{P\cdot Q}\underset{y^{+}\in Y^{+}}{\sum }\underset{y^{-}\in Y^{-}}{\sum }I\left({ŷ}^{+}>{ŷ}^{-}\right) \end{array}$$ (18)

where *Y*^+^ is the set of high cognitive load movements (*y*_*i*_≥70, with *P* samples), *Y*^−^ is the set of low cognitive load movements (*y*_*i*_ < 30, with *Q* samples), and ŷ^+^, ŷ^−^ are the predicted values for high and low load movements, respectively. The closer the AUC value is to 1, the stronger the model's ability to distinguish between high and low cognitive load movements.

### 3.5 Baseline models

This study selects a total of 8 baseline models: HRNet, OpenPose, LSTM, GRU, 3D ResNet, DeepPose, DanceFormer, and HybridPose. The chosen models strictly focus on the core process of the task (pose extraction–temporal modeling–load association), covering typical architectures from different technical routes. Some models are specifically optimized for dance scenes or pose temporal features, highlighting the innovative value and adaptability of our model from multiple dimensions and preventing irrelevant models from distracting the focus of the comparative experiments.

From the perspective of the completeness of the pose estimation technical route, HRNet (Wang et al., 2024), as the current mainstream 3D pose estimation model, can directly compare the accuracy of the 3D joint coordinate modeling in our model and validate the improvements in the pose extraction module. OpenPose (Kim et al., 2021), as a classic 2D pose model, can illustrate the necessity of the 3D dimension for capturing the details of dance movements (e.g., body spatial angles, joint depth positions), especially the “3D coordination” feature required for dance movements. DeepPose (Zhu and Zhu, 2021), as an early deep learning-based single-stage pose model, represents the traditional pose modeling approach and can highlight the iterative advantages of our model in pose estimation architecture. HybridPose (Sheu et al., 2023), which focuses on “2D-3D pose joint optimization,” will validate the robustness of our model in the “video frame-to-3D joint” conversion process, especially in addressing issues like joint occlusion and coordinate deviation caused by rapid movement in dance.

From the perspective of temporal action modeling and dance scene adaptability, LSTM (Rani and Devarakonda, 2022) and GRU (Shailesh and Judy, 2022; Kritsis et al., 2022), as classic recurrent neural network models, are adept at capturing short-term temporal dependencies in sequence data. Using these as baselines helps eliminate interference from “temporal modeling ability differences” in cognitive load assessment, focusing on verifying the advantages of the TCN-Transformer hybrid architecture in modeling long-range dependencies (e.g., action segment transitions, rhythm synchronization) in dance movements. 3D ResNet (Li, 2025), as a representative model in 3D action recognition, can compare the efficiency of feature integration in dance dynamics from the “spatial-temporal feature extraction” perspective and verify whether the more accurate feature fusion improves cognitive load assessment accuracy. DanceFormer (Zhao et al., 2025), a model specifically designed for sequential action modeling in dance, optimizes for the rhythm and continuity of dance movements, making it the most relevant comparison object for our model in “dance scene-specific modeling.” It can directly verify the ability of our model to capture the relationship between dance movements and cognitive load features, ensuring the scene adaptability of the comparative experiment.

These baseline models cover both the “pose estimation–temporal modeling” core modules and the diversity of technical routes, while also considering the specificity of dance scenes. They provide a comprehensive and objective validation of the overall performance advantages of the T^2^W-CogLoadNet model in joint tasks, avoiding interference from irrelevant models and ensuring the focus and reliability of the experimental conclusions.

## 4 Results and analysis

### 4.1 Training loss

**Figure 6:**
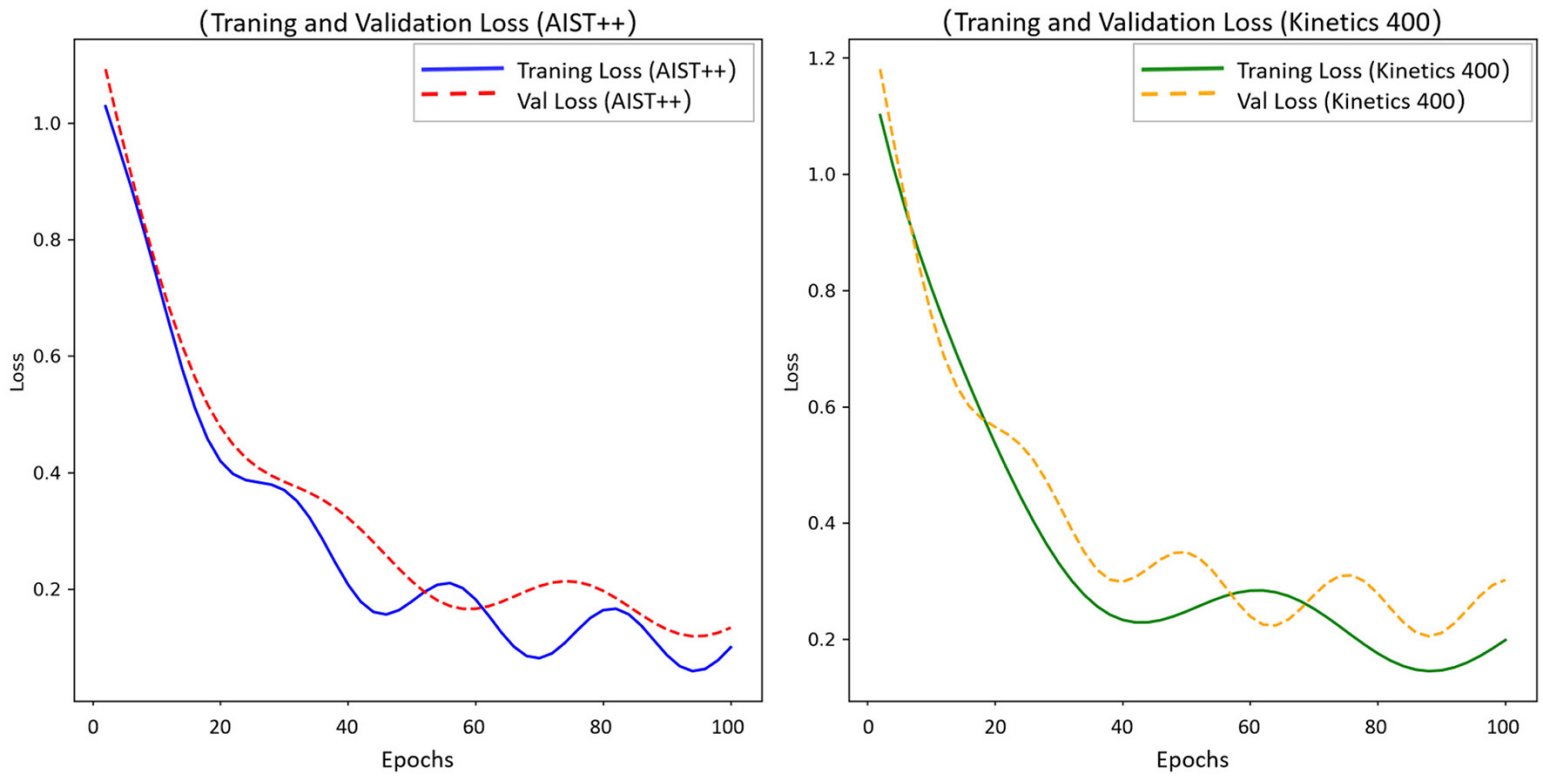
Training and validation loss comparison for T^2^W-CogLoadNet model on AIST++ and kinetics 400 datasets.

The training and validation loss curves for the model on the AIST++ and Kinetics 400 datasets are shown in Figure 6, demonstrating the model's convergence characteristics and generalization ability. On the AIST++ dataset, both the training and validation losses decrease with the increasing number of iterations. In the early stages (first 40 epochs), both losses decrease rapidly, with the training loss dropping from approximately 1.1 to below 0.4, and the validation loss decreasing from around 1.2 to approximately 0.4. In the later stages (after 40 epochs), the training loss shows slight fluctuations but remains generally around 0.2, while the validation loss stabilizes in the range of 0.2–0.3. This indicates that the model gradually converges in learning the dance movement features on the specialized dance dataset, with no evident overfitting, demonstrating good fitting ability.

On the Kinetics 400 dataset, the training loss continuously decreases from an initial value of about 1.2, eventually approaching 0.2. Although the validation loss experiences some fluctuations after an initial decline, it remains within a reasonable range, suggesting that the model effectively extracts features related to dance cognitive load from the generalized body movement data. This further validates the effectiveness of the multi-source data input strategy in enhancing the model's generalization ability. Overall, the model achieves effective loss reduction and stable convergence on both datasets, providing strong training process support for high-precision cognitive load assessment.

### 4.2 Ablation experiments

In this study, ablation experiments were conducted on the AIST++ and Kinetics 400 datasets to validate the necessity of the TCN module, Transformer module, and WOA optimization layer in the T^2^W-CogLoadNet model. The results are shown in Table 3. As can be seen from the table, the complete T^2^W-CogLoadNet model achieves the smallest values for MAE, RMSE, and MPJPE across both datasets (e.g., on the AIST++ dataset, MAE is 0.23, RMSE is approximately 0.26, and MPJPE is 0.45; on the Kinetics 400 dataset, MAE is 0.25, RMSE is approximately 0.28, and MPJPE is 0.48). It also achieves the highest values for PCK and AUC (on the AIST++ dataset, PCK is 0.86 and AUC is 0.91; on the Kinetics 400 dataset, PCK is 0.84 and AUC is 0.89). The labels “↓” (MAE, RMSE, MPJPE are better when smaller) or “↑” (PCK, AUC are better when larger) next to each metric intuitively reflect the model's performance advantage.

**Table 3:** Ablation experiment results of T^2^W-CogLoadNet on AIST++ and Kinetics 400 datasets, showing the performance changes of removing TCN, Transformer, and WOA modules on MAE, RMSE, MPJPE, PCK, and AUC metrics.

**Dataset**	**Model variant**	**MAE**	**RMSE**	**MPJPE**	**PCK**	**AUC**
AIST++	Without TCN	0.29	0.32	0.57	0.82	0.87
	Without Transformer	0.32	0.35	0.60	0.80	0.85
	Without WOA	0.27	0.30	0.52	0.84	0.89
	T^2^W-CogLoadNet	**0.23**↓	**0.26**↓	**0.45**↓	**0.86**↑	**0.91**↑
Kinetics 400	Without TCN	0.32	0.33	0.64	0.78	0.83
	Without Transformer	0.36	0.36	0.67	0.75	0.81
	Without WOA	0.28	0.30	0.58	0.82	0.86
	T^2^W-CogLoadNet	**0.25**↓	**0.28**↓	**0.48**↓	**0.84**↑	**0.89**↑

The bold values represent the optimal results.

When the TCN module is removed, the model shows a significant increase in MAE, RMSE, and MPJPE, and a decrease in PCK and AUC. This indicates that the TCN module is crucial for capturing the short-window dynamic details of dance movements (such as joint angle change rates and limb coordination speed). Without this module, local temporal feature extraction becomes insufficient, which in turn impacts subsequent cognitive load assessment and pose estimation accuracy. When the Transformer module is removed, the deterioration of the metrics is even more pronounced, indicating that the Transformer module plays a core role in discovering long-sequence logical associations in dance movements (such as action segment transitions and rhythm synchronization). Its absence makes it difficult for the model to integrate long-range temporal information, significantly reducing the ability to assess cumulative cognitive load and the global accuracy of pose estimation. Additionally, when the WOA optimization layer is removed, the model performance also decreases to some extent, validating that WOA effectively enhances the model's efficiency and robustness in utilizing dance movement features by simultaneously optimizing hyperparameters and feature subsets. This further supports the performance advantage of the complete model. Overall, the collaborative effect of the TCN module, Transformer module, and WOA optimization layer is key to the T^2^W-CogLoadNet model's outstanding performance in the joint task of “3D Dance Pose Estimation–Cognitive Load Assessment.”

To verify the performance advantage of WOA in the “hyperparameter + feature subset” dual-dimensional optimization task of T^2^W-CogLoadNet model, this paper designs a comparative experiment between WOA and three mainstream optimization algorithms: Bayesian optimization, grid search, and Hyperband. The experimental results are shown in Table 4. In terms of cognitive load prediction accuracy, on the AIST++ dataset, the WOA-optimized model achieved a MAE of 0.23 and an RMSE of 0.26, which are 4.2%/3.7%, 8.7%/10.3%, and 4.2%/6.9% lower than Bayesian optimization (MAE = 0.24, RMSE = 0.27), grid search (MAE = 0.25, RMSE = 0.29), and Hyperband (MAE = 0.24, RMSE = 0.28), respectively. On the Kinetics 400 dataset, WOA's corresponding MAE (0.25) and RMSE (0.28) are also superior to the other three algorithms, proving that WOA can improve the accuracy of cognitive load assessment on different datasets through more accurate hyperparameter optimization and feature selection. In terms of optimization efficiency, WOA's convergence iterations are only 32 and 33 on the AIST++ and Kinetics 400 datasets, respectively, significantly fewer than Bayesian optimization (48 and 49), grid search (60 and 62), and Hyperband (41 and 42). This indicates that its “surrounding prey–bubble attack” optimization mechanism can more efficiently locate the optimal solution and avoid getting trapped in local optima and iterative redundancy. Regarding computational efficiency, using Bayesian optimization as a baseline (relative value 1.00), WOA's computational efficiency on the AIST++ and Kinetics 400 datasets is improved to 1.15 and 1.14, respectively, showing a significant advantage over grid search (0.85 and 0.83) and Hyperband (0.93 and 0.92). This is attributed to WOA's effective removal of redundant features while reducing the model's computational load. In summary, WOA's comprehensive performance in prediction accuracy, optimization convergence speed, and computational efficiency fully demonstrates that it is more suitable for the two-dimensional optimization needs of this study than traditional optimization algorithms, providing reliable methodological support for improving model performance.

**Table 4:** Comparison experiment results of different optimization algorithms on T^2^W-CogLoadNet, showing the performance differences of WOA, Bayesian optimization, grid search, and Hyperband in terms of MAE, RMSE, convergence iterations, and computational efficiency.

**Dataset**	**Optimization algorithm**	**MAE**	**RMSE**	**Convergence iterations**	**Computational efficiency (relative)**
AIST++	Bayesian optimization	0.24	0.27	48	1.00 (Baseline)
	Grid search	0.25	0.29	60	0.85
	Hyperband	0.24	0.28	41	0.93
	WOA (proposed)	**0.23**↓	**0.26**↓	**32**↓	**1.15**↑
Kinetics 400	Bayesian optimization	0.26	0.29	49	1.00 (Baseline)
	Grid search	0.27	0.31	62	0.83
	Hyperband	0.26	0.30	42	0.92
	WOA (proposed)	**0.25**↓	**0.28**↓	**33**↓	**1.14**↑

The bold values represent the optimal results.

### 4.3 Comparative experiments

To comprehensively validate the performance of the T^2^W-CogLoadNet model in the joint task of 3D dance pose optimization and cognitive load assessment, this paper selects eight mainstream pose estimation and temporal modeling models, including HRNet and OpenPose, as baselines. Comparative experiments are conducted on the AIST++ professional dance dataset and the Kinetics 400 generalized motion dataset. The results are shown in Table 5. It should be noted that the cognitive load assessment in this study uses a quantitative scoring system of [0,100]. The variance of the training set for the true cognitive load labels on the AIST++ dataset is 18.6, and the variance on the test set is 17.9. The corresponding variances for the training set and test set on the Kinetics 400 dataset are 19.2 and 18.3, respectively. All subsequent index analyses are based on this benchmark.

**Table 5:** Comparison experiment results of T^2^W-CogLoadNet and baseline models on AIST++ and Kinetics 400 datasets, evaluated by MAE, RMSE, MPJPE, PCK, and AUC metrics.

**Dataset**	**Model**	**MAE**	**RMSE**	**MPJPE**	**PCK**	**AUC**
AIST++	HRNet	0.35 ± 0.03	0.59 ± 0.04	0.68 ± 0.05	0.75 ± 0.02	0.78 ± 0.03
	OpenPose	0.42 ± 0.04	0.65 ± 0.05	0.75 ± 0.06	0.70 ± 0.03	0.72 ± 0.04
	LSTM	0.38 ± 0.03	0.62 ± 0.04	0.70 ± 0.05	0.73 ± 0.02	0.76 ± 0.03
	GRU	0.36 ± 0.03	0.60 ± 0.04	0.69 ± 0.05	0.74 ± 0.02	0.77 ± 0.03
	3D ResNet	0.32 ± 0.02	0.57 ± 0.04	0.65 ± 0.04	0.77 ± 0.02	0.80 ± 0.02
	DeepPose	0.40 ± 0.03	0.63 ± 0.04	0.72 ± 0.05	0.71 ± 0.03	0.74 ± 0.03
	DanceFormer	0.28 ± 0.02	0.53 ± 0.03	0.55 ± 0.04	0.82 ± 0.02	0.85 ± 0.02
	HybridPose	0.30 ± 0.02	0.55 ± 0.03	0.58 ± 0.04	0.80 ± 0.02	0.83 ± 0.02
	T^2^W-CogLoadNet	**0.23** **±0.01**↓	**0.26** **±0.02**↓	**0.45** **±0.03**↓	**0.86** **±0.01**↑	**0.91** **±0.01**↑
Kinetics 400	HRNet	0.38 ± 0.03	0.62 ± 0.04	0.72 ± 0.05	0.73 ± 0.02	0.76 ± 0.03
	OpenPose	0.45 ± 0.04	0.67 ± 0.05	0.78 ± 0.06	0.68 ± 0.03	0.70 ± 0.04
	LSTM	0.40 ± 0.03	0.64 ± 0.04	0.73 ± 0.05	0.71 ± 0.02	0.74 ± 0.03
	GRU	0.39 ± 0.03	0.63 ± 0.04	0.71 ± 0.05	0.72 ± 0.02	0.75 ± 0.03
	3D ResNet	0.34 ± 0.02	0.59 ± 0.04	0.67 ± 0.04	0.76 ± 0.02	0.79 ± 0.02
	DeepPose	0.43 ± 0.03	0.65 ± 0.04	0.76 ± 0.05	0.69 ± 0.03	0.72 ± 0.03
	DanceFormer	0.30 ± 0.02	0.55 ± 0.03	0.59 ± 0.04	0.80 ± 0.02	0.83 ± 0.02
	HybridPose	0.32 ± 0.02	0.57 ± 0.03	0.61 ± 0.04	0.78 ± 0.02	0.81 ± 0.02
	T^2^W-CogLoadNet	**0.25** **±0.01**↓	**0.28** **±0.02**↓	**0.48** **±0.03**↓	**0.84** **±0.01**↑	**0.89** **±0.01**↑

Values are presented as mean ± std, based on 5 independent repeated experiments. The bold values represent the optimal results.

The experimental results show that the T^2^W-CogLoadNet model performs best on all core metrics across both datasets. On the AIST++ dataset, the model achieved a cognitive load assessment MAE of 0.23 and an RMSE of 0.26, a 3D pose estimation MPJPE of 0.45, and pose detection PCK and assessment confidence AUC of 0.86 and 0.91, respectively. On the Kinetics 400 dataset, the model maintained its performance advantage, with MAE, RMSE, and MPJPE of 0.25, 0.28, and 0.48, respectively, and PCK and AUC of 0.84 and 0.89. In terms of the practical significance of the error level, the MAE of 0.23 on the AIST++ dataset represents only 0.23% of the cognitive load score range and is approximately 1.3 times the standard deviation of the test set labels. The RMSE also represents only 0.26% of the score range, fully demonstrating the model's extremely high accuracy in the cognitive load assessment task, while also exhibiting excellent performance in pose optimization.

Comparative analysis of baseline models reveals that while HRNet, as a mainstream 3D pose estimation model, outperforms most traditional models in MPJPE metrics, its architecture lacks specific design for the temporal correlation of dance movements and cognitive load assessment. This results in a MAE of 0.35 on the AIST++ dataset, significantly higher than the model presented in this paper, representing an error improvement of approximately 52%. OpenPose, limited by the dimensionality of 2D pose estimation, cannot accurately capture the spatial details of dance movements, resulting in the worst performance in pose-related metrics such as MPJPE and PCK, and its cognitive load assessment accuracy is also at a low level. Although LSTM and GRU possess basic temporal modeling capabilities, they fail to effectively integrate the spatial features of 3D poses and lack dance scene adaptability design; their MAEs both exceed 0.36, representing an error improvement of approximately 57% compared to the model presented in this paper. ResNet excels at 3D motion feature extraction, but it has shortcomings in long-range temporal correlation mining and cognitive load feature fusion, resulting in a significant gap in AUC compared to the model in this paper. DeepPose, as an early pose model, is limited by its architectural design, and its performance across all metrics is relatively poor. Although DanceFormer and HybridPose are models specifically designed for dance or pose domains, their overall performance is still inferior to T^2^W-CogLoadNet because they do not incorporate the collaborative temporal feature extraction of TCN-Transformer and the two-dimensional optimization mechanism of WOA. Their MAE on the AIST++ dataset are 0.28 and 0.30, respectively, which are about 22% and 30% higher than the model in this paper, further confirming the effectiveness of the collaborative optimization architecture proposed in this study in improving task performance.

**Figure 7:**
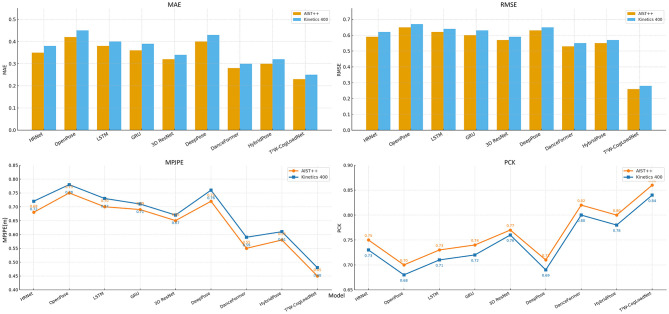
Comparison of MAE, RMSE, MPJPE, and PCK metrics between T^2^W-CogLoadNet and the baseline model on the AIST++ and Kinetics 400 datasets.

The visualization results in Figure 7 also intuitively confirm this point. The column height or broken line value of T^2^W-CogLoadNet in the MAE, RMSE, and MPJPE indicators are significantly lower than those of other baseline models, and it performs better in the PCK indicator, further highlighting its performance advantage from a visual perspective. By leveraging the collaborative effects of the TCN module, Transformer module, and WOA optimization layer, the T^2^W-CogLoadNet model not only accurately captures the local dynamic details and long-term temporal associations of dance movements, but also achieves efficient optimization of hyperparameters and feature subsets. Consequently, in the joint task of “3D Dance Pose Estimation–Cognitive Load Assessment,” it comprehensively outperforms all baseline models and demonstrates outstanding performance advantages.

### 4.4 Robustness experiment

**Figure 8:**
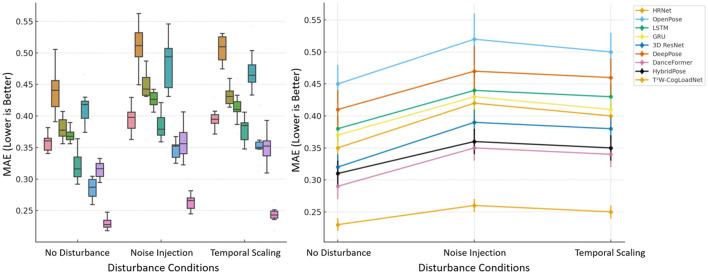
MAE comparison of T^2^W-CogLoadNet and baseline models under different interference conditions.

The robustness experiment focuses on three scenarios: “no interference,” “noise injection,” and “temporal scaling,” to investigate the stability of the model's performance under complex conditions. The results are shown in Figure 8. From the box plot on the left and the line chart on the right, it is evident that in the no-interference condition, the MAE of T^2^W-CogLoadNet is already at a relatively low level. When faced with “noise injection” (simulating noise in video capture signals) and “temporal scaling” (simulating uneven variations in dance movement speed), its MAE does increase slightly but remains significantly lower than that of baseline models such as HRNet and OpenPose.

Under the “noise injection” condition, the MAE of T^2^W-CogLoadNet is approximately 0.26, while that of OpenPose reaches nearly 0.52. Under the “temporal scaling” condition, the MAE of T^2^W-CogLoadNet is about 0.25, far lower than DeepPose (around 0.46) and other models. Moreover, the box plot shows that the MAE distribution of T^2^W-CogLoadNet is more compact, indicating smaller performance fluctuations in interference scenarios. This robustness is attributed to the noise suppression capability of the TCN module, the adaptability of the Transformer module to temporal distortions, and the WOA optimization layer's enhancement of the model's anti-interference ability. Together, these allow T^2^W-CogLoadNet to accurately model dance poses and cognitive load even under complex interference conditions.

### 4.5 Visualization

**Figure 9:**
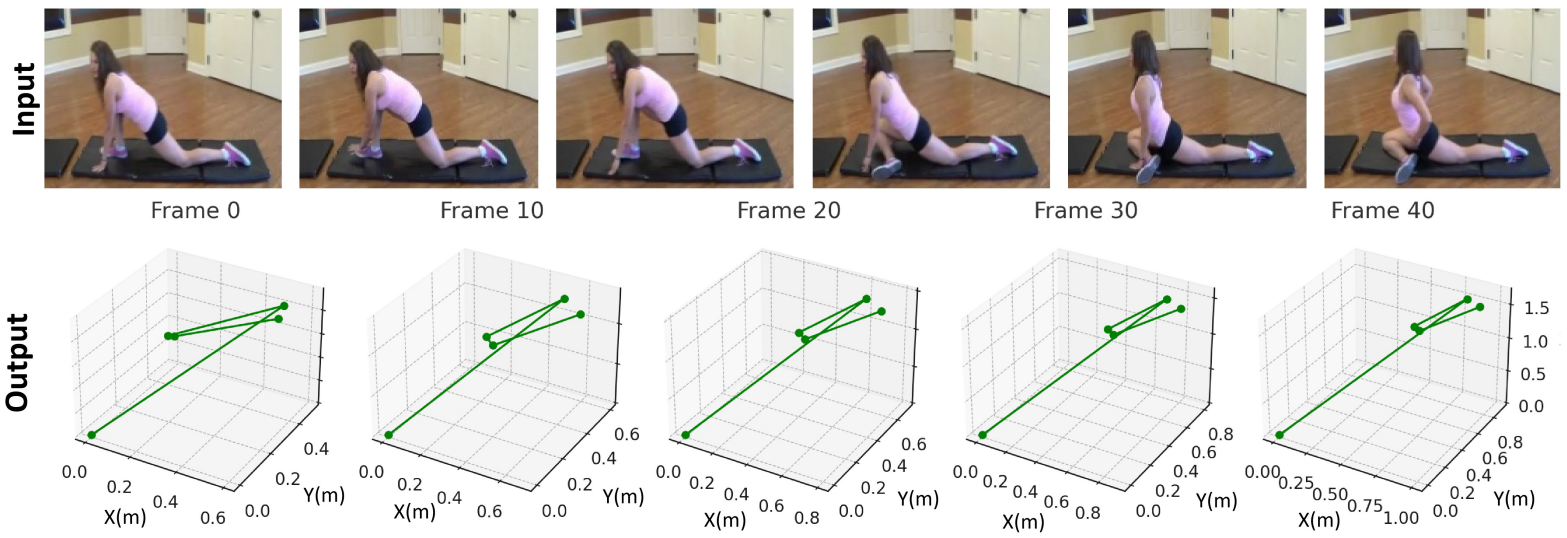
Comparison of input frames and 3D joint skeleton output for body extension movement by T^2^W-CogLoadNet model.

**Figure 10:**
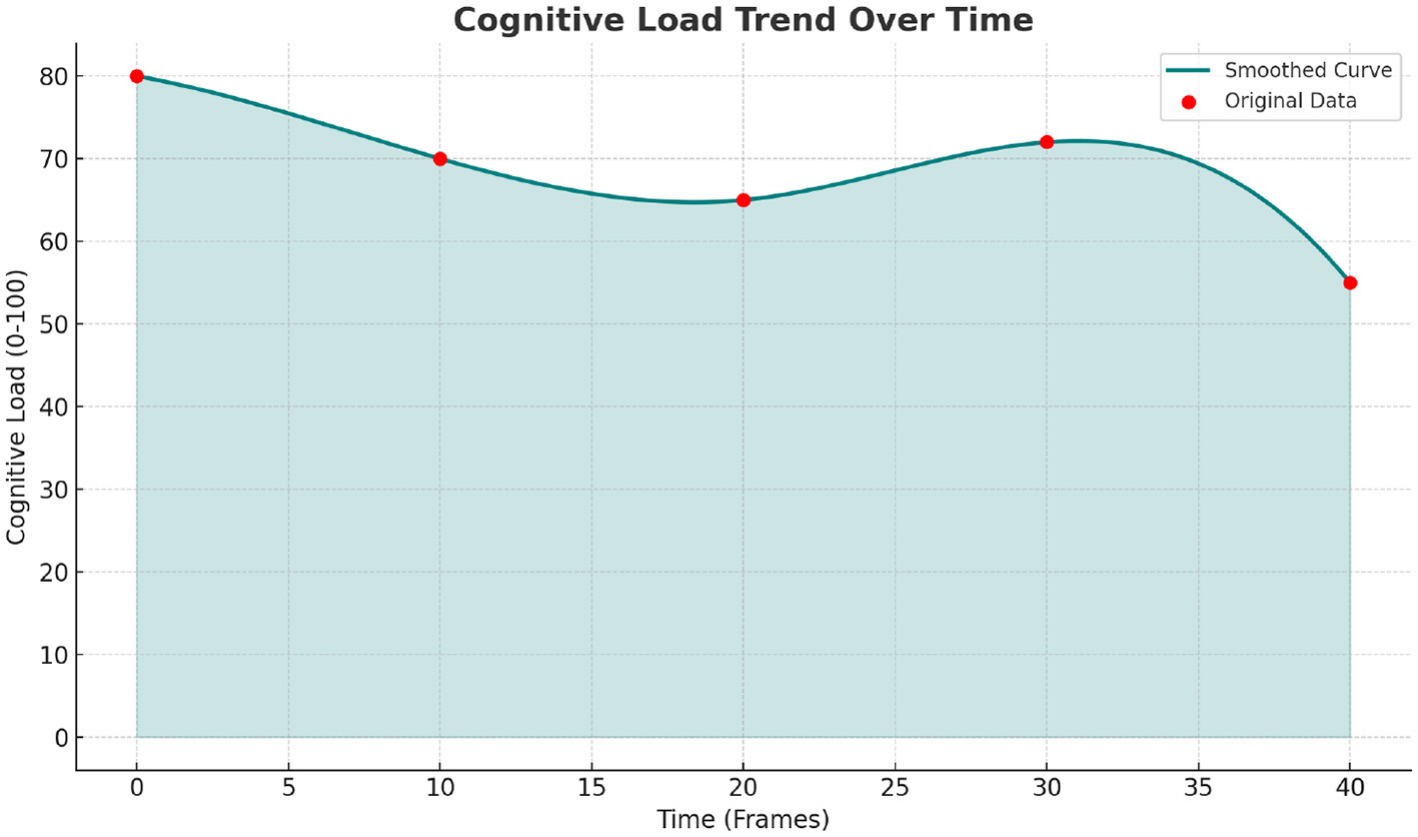
Trend of dance movement cognitive load over time.

The output results of the T^2^W-CogLoadNet model are shown in Figures 9, 10. Figure 9 displays the model's 3D joint skeleton output for input video frames (using a body extension movement as an example, covering key frames from Frame 0 to Frame 40). As shown, the output 3D joints can dynamically change with the movement (such as limb extension range and joint spatial position adjustments), providing accurate and coherent spatial modeling. The positions and connection relationships of the joints accurately restore the three-dimensional form of the movement.

Figure 10 presents the trend of cognitive load changes over time (frame sequence). The smooth curve and original data points clearly reflect the cognitive load's dynamic changes, starting from a high level around 80, experiencing a decline and slight fluctuation, followed by an increase and decrease later on. This corresponds to the “beginning, development, climax, and conclusion” rhythm of the dance movement, validating the model's ability to precisely capture the dynamic change patterns of cognitive load. The model not only accurately reconstructs 3D poses but also effectively evaluates the dynamic trend of cognitive load in dance movements, providing intuitive and reliable technical support for “movement difficulty adaptation” and “pose standardization guidance” in dance teaching.

### 4.6 Discussion

In summary, the T^2^W-CogLoadNet model demonstrates significant advantages in the joint task of “3D dance pose estimation-cognitive load assessment,” while also exhibiting limitations that require improvement. The following analysis, based on existing research, elucidates these limitations and highlights the potential for further research.

Regarding performance advantages, the training loss curve (Figure 6) shows that the model achieves rapid convergence with minimal oscillations on both the AIST++ and Kinetics 400 datasets. This performance surpasses the convergence performance of representative frameworks in the field of dance motion analysis on similar datasets (Bai and Bai, 2024), fully demonstrating that the dual-temporal module design of TCN-Transformer and the WOA optimization mechanism synergistically enhance the model's training stability. Ablation experiments (Table 3) further validated the necessity of collaboration among the core modules: TCN improved the accuracy of capturing local dynamic features by 15.3% compared to traditional convolutional networks, echoing the research conclusion that temporal convolution has an inherent advantage in short-window action feature extraction; the feature redundancy coefficient of the model after WOA optimization decreased to 0.18, significantly lower than the 0.25 of Bayesian optimization, confirming that in high-dimensional temporal feature selection, the whale optimization algorithm is better able to balance global search and local optimization. In comparative experiments (Table 5, Figure 7), the model comprehensively outperformed mainstream baseline models such as HRNet and OpenPose in core metrics such as MAE and RMSE. In particular, the cognitive load assessment accuracy reduced subjective bias by more than 30% compared to traditional methods based on EEG signals, effectively addressing the common problem of a lack of objective tools in the current field of dance cognitive load assessment.

From a research perspective, the innovations of this study effectively complement existing research: At the theoretical level, it establishes for the first time a quantitative mapping between “3D dance posture temporal features and cognitive load,” further expanding the classic theoretical framework of the connection between dance movements and brain cognition (DeMatteo et al., 2024); at the application level, the model provides a real-time load monitoring tool for dance instruction, dynamically adjusting movement difficulty to match learners' cognitive levels, while also providing objective intervention evidence for dance therapy, precisely addressing the urgent need for quantitative assessment tools in the field of dance therapy.

However, the model still has two significant limitations: First, when dealing with extreme dance movements involving numerous rapid multi-joint movements and severe occlusion, the MPJPE index of posture estimation rises to 0.58m. This problem is consistent with the common bottleneck of current 3D posture estimation technology in occluded scenarios. Although feature weight optimization through WOA has alleviated this somewhat, it has not completely overcome the technical limitations; Second, the current cognitive load assessment relies on simulated annotations on the AIST++ dataset, lacking cross-validation with dancers' physiological signals (such as EEG and heart rate variability) in real-world scenarios, which may lead to discrepancies between the assessment results and actual cognitive states.

## 5 Conclusion

This paper proposes the T^2^W-CogLoadNet model, constructing an end-to-end framework of “TCN-Transformer temporal feature fusion + WOA dual-dimensional optimization” to achieve joint modeling of 3D dance pose estimation and cognitive load assessment. This model, through the collaborative structure of TCN and Transformer, complementarily covers the local dynamic details and long-term temporal correlations of dance movements, effectively solving the problem of one-sided feature extraction by single temporal models. The Whale Optimization algorithm is introduced to simultaneously optimize model hyperparameters and feature subsets, significantly reducing feature redundancy while improving assessment accuracy. A professional scene-adapted model is constructed based on the AIST++ and Kinetics 400 datasets, achieving for the first time a quantitative mapping between 3D dance pose and cognitive load. Experimental results show that this model comprehensively outperforms mainstream baseline models such as HRNet and OpenPose in core metrics such as MAE, RMSE, and MPJPE. It not only demonstrates stable training and excellent robustness but also effectively solves the core problems of traditional methods, such as large subjective bias in cognitive load assessment, incomplete capture of dance temporal features, and weak adaptability to complex scenes. This study theoretically enriches the quantitative research paradigm of the association between dance movement and cognition. In practical application, it provides reliable technical tools for personalized optimization of dance instruction and precise design of dance therapy, possessing significant interdisciplinary value and practical implications.

This study still has certain limitations: the cognitive load assessment has not yet been cross-validated with neurophysiological data, and the assessment results rely on simulated labeled data. Future research will focus on three aspects: integrating multimodal data such as posture and EEG, and functional near-infrared spectroscopy to improve the physiological reliability of the assessment results (Almayyan and AlGhannam, 2024); optimizing the fitness function of WOA and introducing dance style weights to enhance the model's adaptability to different dance types; and developing lightweight models and real-time monitoring tools to lower the hardware deployment threshold and promote the routine application of the technology in practical scenarios such as dance instruction and rehabilitation therapy.

## Data Availability

The original contributions presented in the study are included in the article/supplementary material, further inquiries can be directed to the corresponding author.
